# SIRT1 Promotes M2 Microglia Polarization *via* Reducing ROS-Mediated NLRP3 Inflammasome Signaling After Subarachnoid Hemorrhage

**DOI:** 10.3389/fimmu.2021.770744

**Published:** 2021-11-24

**Authors:** Da-Yong Xia, Jin-Long Yuan, Xiao-Chun Jiang, Min Qi, Nian-Sheng Lai, Ling-Yun Wu, Xiang-Sheng Zhang

**Affiliations:** ^1^ Department of Neurosurgery, The First Affiliated Hospital of Wannan Medical College (Yijishan Hospital of Wannan Medical College), Wuhu, China; ^2^ Department of Neurosurgery, Nanjing Drum Tower Hospital, The Affiliated Hospital of Nanjing University Medical School, Nanjing, China; ^3^ Department of Neurosurgery, Beijing Friendship Hospital, Capital Medical University, Beijing, China

**Keywords:** microglia polarization, early brain injury, subarachnoid hemorrhage, sirtuin 1, NLRP3

## Abstract

Mounting evidence has suggested that modulating microglia polarization from pro-inflammatory M1 phenotype to anti-inflammatory M2 state might be a potential therapeutic approach in the treatment of subarachnoid hemorrhage (SAH) injury. Our previous study has indicated that sirtuin 1 (SIRT1) could ameliorate early brain injury (EBI) in SAH by reducing oxidative damage and neuroinflammation. However, the effects of SIRT1 on microglial polarization and the underlying molecular mechanisms after SAH have not been fully illustrated. In the present study, we first observed that EX527, a potent selective SIRT1 inhibitor, enhanced microglial M1 polarization and nod-like receptor pyrin domain-containing 3 (NLRP3) inflammasome activation in microglia after SAH. Administration of SRT1720, an agonist of SIRT1, significantly enhanced SIRT1 expression, improved functional recovery, and ameliorated brain edema and neuronal death after SAH. Moreover, SRT1720 modulated the microglia polarization shift from the M1 phenotype and skewed toward the M2 phenotype. Additionally, SRT1720 significantly decreased acetylation of forkhead box protein O1, inhibited the overproduction of reactive oxygen species (ROS) and suppressed NLRP3 inflammasome signaling. In contrast, EX527 abated the upregulation of SIRT1 and reversed the inhibitory effects of SRT1720 on ROS-NLRP3 inflammasome activation and EBI. Similarly, *in vitro*, SRT1720 suppressed inflammatory response, oxidative damage, and neuronal degeneration, and improved cell viability in neurons and microglia co-culture system. These effects were associated with the suppression of ROS-NLRP3 inflammasome and stimulation of SIRT1 signaling, which could be abated by EX527. Altogether, these findings indicate that SRT1720, an SIRT1 agonist, can ameliorate EBI after SAH by shifting the microglial phenotype toward M2 *via* modulation of ROS-mediated NLRP3 inflammasome signaling.

## Introduction

Subarachnoid hemorrhage (SAH) is a fatal cerebral vascular disease with high morbidity and mortality worldwide. Despite tremendous advances in diagnosis and neurosurgical care, it remains a difficult challenge to develop effective therapeutic interventions to improve SAH outcomes. The etiology of SAH is complicated. Accumulating evidence suggests that the robust post-SAH inflammatory response plays a crucial role in causing early brain injury (EBI) after SAH (1–3). With the occurrence of hemorrhage and ischemia, rapid influx of inflammatory cells, microglia activation, and an increase of proinflammatory cytokines production lead to neuronal cell death. In addition, inflammatory cells could induce the generation of reactive oxygen species (ROS) to further aggravate EBI. Therefore, diminishing cerebral inflammation is a promising method to reduce EBI and improve brain recovery after SAH.

As key innate immune cells, microglia play critical roles in the innate immune response and exert distinct tissue repair functions after SAH (4). Microglia can sense slight imbalances in immune homeostasis, and cytotoxic mediators and endogenous proteins released from damaged neural cells stimulate resting microglia activation (5). In response to acute brain injury, activated microglia can develop into either classically M1 (proinflammatory) or alternatively activated M2 (anti-inflammatory) phenotypes (6). For example, in a mouse model of spinal cord injury (SCI), both M1 and M2 marker levels were increased in the acute phase. But the flow cytometry results indicated that microglia and macrophages had mainly developed an M1 phenotype (7). In models of traumatic brain injury (TBI) and ischaemic stroke, a dynamic change of M2 to M1 phenotype was observed. In time, the M1 microglia/macrophages become dominant in the injury area and aggravate brain damage by exacerbating inflammatory response (8, 9). In SAH area, numerous studies also reported that the M1 microglia/macrophages was predominant in the early phase and subsequently transited to the M2 phenotype. Inhibition of microglia M1 polarization and promotion of a shift from M1 to M2 phenotype significantly ameliorated neuroinflammation and improved neurological outcomes after SAH (10–12). Thus, targeting the microglial phenotypic transformation might provide a promising means to reduce EBI after SAH.

SIRT1, a member of the sirtuin family, plays a prominent role in regulating various biological functions, such as immune response, apoptosis, oxidative stress, and aging (3, 13). The function of SIRT1 is increasingly recognized as especially important in the pathogenesis of neurological diseases. Accumulating studies have demonstrated that SIRT1 plays a protective role in various models of neuronal injury and neurodegeneration (14–16). For example, in a permanent focal ischemia mice model, SIRT1 plays a key role in neuroprotection against brain ischemia by inhibition of inflammatory and apoptotic pathways (17). In models of TBI, SIRT1 activation ameliorates oxidative damage and neuronal apoptosis by modulation of nuclear factor-erythroid 2-related factor 2 (Nrf2) signaling pathway (18). Meanwhile, emerging evidence has indicated that SIRT1 is involved in and plays a pivotal role in the pathophysiology of SAH. Vellimana et al. reported that SIRT1 inhibition blocked the protective effects of hypoxic preconditioning on vasospasm and neurological deficits after SAH. In contrast, SIRT1 activation by resveratrol pretreatment abrogated vasospasm and attenuated neurological deficits following SAH (19, 20). Another two previous studies observed that SIRT1 inhibition by sirtinol aggravated brain edema, oxidative damage, neuroinflammation, and neuronal apoptosis after SAH (13, 21). However, it is not clear whether SIRT1 activation modulates the phenotypes and functions of activated microglia after SAH as well as the potential mechanisms. Previous studies have reported that SIRT1 participates in regulating immune responses and inducing M2 microglia in a variety of neurological disorders (22, 23). For example, Fu et al. showed that SIRT1 activation could modulate the microglia polarization shift from the M1 phenotype and skew toward the M2 phenotype in a cerebral ischemic/reperfusion injury model (24). Duan et al. indicated that activation of SIRT1 ameliorated chronic unpredictable mild stress-induced depressive-like behaviors *via* shifting microglial polarization toward the M2 phenotype (22). Therefore, we hypothesized that SIRT1 might modulate microglial phenotypic transformation to protect against EBI after SAH.

The nod-like receptor pyrin domain-containing protein 3 (NLRP3) inflammasome is a key mediator of numerous vascular diseases through triggering a series of immune-inflammatory response (25). It has been demonstrated that the NLRP3 inflammasome is expressed in microglia and implicated in the modulation of inflammatory responses by switching microglia M1/M2 phenotypes (26, 27). Intriguingly, emerging evidence indicates that SIRT1 could regulate NLRP3 inflammasome signaling in different research fields (3, 28, 29). However, the relationship between SIRT1 and NLRP3 inflammasome signaling in modulation of microglia polarization after SAH is still unknown. Based on these backgrounds, we sought to evaluate the effects of SIRT1 activation on microglia polarization and investigate the relationship between SIRT1 and NLRP3 inflammasome signaling in a SAH model.

## Methods and Materials

All procedures were approved by the Animal Care and Use Committee of Wannan Medical College and conformed to the *Guide for the Care and Use of Laboratory Animals* published by the National Institutes of Health.

### Animals and SAH Model

Adult male Sprague-Dawley rats (250-300 g) were bought from the Nanjing Biomedical Research Institute of Nanjing University. A prechiasmatic cistern injection models was built according to our previous study (13). Briefly, after anesthetization with avertin (200 mg/kg), rats were fixed in a stereotactic apparatus. The skin overlaying the anterior skull was opened, and a hole was drilled in the midline 7.5 mm anterior to the bregma. Blood (0.35 mL) from the femoral artery was injected through the hole over 20 s. Sham animals received the same procedure with injection of 0.35 ml of physiologic saline instead of blood.

### *In Vivo* Study Design

In the first set of experiments, 48 rats (64 rats were used, 16 rats died) were divided into the following groups: SAH + vehicle (n= 15, 3 rats died), SAH+5 mg/kg EX527 (n= 16, 4 rats died), SAH + 10 mg/kg EX527 (n= 16, 4 rats died), SAH + 15 mg/kg EX527 (n= 17, 5 rats died) groups. Rats were killed at 24 h after SAH. Post-assessments included behavior performance, western blot, and histopathological study.

In the second set of experiments, a total of 142 rats (162 rats were used, 20 rats died) were randomly assigned into sham + vehicle (n= 28), SAH + vehicle (n= 34, 6 rats died), SAH + 50 mg/kg SRT1720 (n= 15, 3 rats died), SAH + 150 mg/kg SRT1720 (n= 32, 4 rats died), SAH + 250 mg/kg SRT1720 group (n= 14, 2 rats died), and SAH + 150 mg/kg SRT1720 + EX527 group (n=39, 5 rats died). Rats were killed at 24 h and 72 h after SAH. Post-assessments included neurological scores, western blotting, biochemical estimation, immunofluorescence staining, histopathological study, and brain water content analysis.

### Primary Cell Culture

Primary neural cell culture was conducted from the cortex of neonatal rats according to previous studies (12, 30). For primary neurons culture, neuronal cells were cultured onto poly-D-lysine –coated plates and suspended in neurobasal media supplemented with B27, glutamate, Hepes, penicillin and streptomycin. For primary microglia culture, cortical cells were suspended in serum-free DMEM-F12 culture medium. The purity of primary microglia was more than 90% in our study (**Supplementary Figure 1**). Regarding the neurons and microglia co-culture system, microglia were seeded in transwell upper chamber and the neurons were cultured in the plates. Co-culture medium was DMEM with 10% FBS. The co-culture system was harvested 24 h after indicated intervention.

For the *in vitro* SAH model, the co-culture system was stimulated with oxyhemoglobin (OxyHb, Bio Basic Inc., USA). The neuron-microglia co-cultures were divided into the following groups: control, OxyHb, OxyHb + SRT1720 (1, 5, and 10 μM), and OxyHb + SRT1720 + EX527. The culture medium and cells were collected for, ELISA, immunofluorescence staining, and cell viability analysis.

### Drug Administration

For *in vivo*, SRT1720 (Selleck) was prepared in 1% dimethyl sulfoxide (DMSO) in physiologic saline before use. SRT1720 or vehicle was administered intraperitoneally at 2 h after surgery and then once a day until euthanasia. EX527 (Sigma-Aldrich, St. Louis, MO, USA) was prepared in 1% (DMSO). EX527 or vehicle was administered intraperitoneally for 3 days before SAH construction. *In vitro*, SRT1720 was dissolved in culture medium. EX527 was dissolved in 1% DMSO (in physiologic saline) and then added to culture medium to reach a final concentration of 20 μM. The doses of SRT1720 and EX527 were selected according to previous studies (31–33).

### Detection of ROS Fluorescence

As previously reported (18), primary neurons were incubated with 2,7-dichlorodihydrofluorescein diacetate (DCFH, Sigma) for 10 min at 37°C. DCFH fluorescence was measured under an inverted fluorescence microscope. The mean relative fluorescence intensity for each group was measured with the Image-Pro Plus system.

### Cell Viability Analysis

The lactate dehydrogenase (LDH) and Cell Counting Kit-8 (CCK-8) kits (Beyotime Biotechnology, China) were used to evaluate the viability of primary cultured neurons according to the manufacturer’s instructions.

### ELISA

Brain samples and culture medium were collected. The levels of IL-1β, tumor necrosis factor-α (TNF-α), IL-6, IL-18, ICAM-1, and CCL-2 were measured with ELISA kits according to the manufacturer instructions (Multi Sciences. China).

### Biochemical Estimation

The levels of intracellular malondialdehyde (MDA), superoxide dismutase (SOD), and glutathione peroxidase (GSH-Px) were evaluated with commercially available kits (Nanjing Jiancheng Bioengineering Institute, Nanjing, China) in accordance with the manufacturer’s instructions.

### Western Blotting

Western blotting was performed according to our previous study (18). After sample preparation, equal amounts of protein samples were separated by polyacrylamide gel electrophoresis and transferred to a polyvinylidene difluoride membrane. The membrane was then incubated with primary antibodies against SIRT1 (1:200, cat# SC-15404, Santa Cruz), ac-FoxO1 (1:200, cat# sc-49437, Santa Cruz), NLRP3 (1:200, cat# SC-66846, Santa Cruz Biotechnology), adaptor apoptosis-related speck-like protein (ASC) (1:200, cat# SC-22514, Santa Cruz Biotechnology), caspase-1 (1:200, cat# SC-56036, Santa Cruz Biotechnology), caspase-1 p20 (1:200, cat# SC-398715, Santa Cruz Biotechnology), and β-actin (1:3000, cat# AP0060, Bioworld Technology, Minneapolis, MN, USA) overnight at 4°C. Subsequently, the membrane was incubated with horseradish peroxidase (HRP)-conjugated IgG for 2 h at room temperature. Protein bands were incubated with enhanced chemiluminescence solution (Thermo Fisher Scientific, Waltham, MA, USA). Band density was analyzed using Image J software.

### Immunohistochemical Staining

Immunohistochemical staining was conducted as previously described (34). In brief, brain sections were fixed with 4% paraformaldehyde and embedded in paraffin. After being deparaffinized, the brain sections were incubated overnight at 4°C with primary antibodies against SIRT1 (1:50, Santa Cruz Biotechnology), NLRP3 (1:50, Santa Cruz Biotechnology), ASC (1:50, Santa Cruz Biotechnology), and caspase-1 p20 (1:50, Santa Cruz Biotechnology). After sections were washed with PBS, they were incubated with HRP-conjugated IgG for 60 min at room temperature. Slides were visualized by incubation with diaminobenzidine and hydrogen peroxide. Staining intensity was scored on a 0-to-4 scale system. In this system, 0 indicates no detectable positive cells and 4 indicates the highest density of positive cells.

### Immunofluorescence Staining and TUNEL Staining

Immunofluorescence staining was performed as previously described (34, 35). Briefly, brain sections (6 μm) were fixed with 4% paraformaldehyde. The sections were incubated overnight at 4°C with primary antibodies against Iba-1 (1:50, cat# SC-98468, Santa Cruz Biotechnology), NLRP3 (1:50, cat# SC-66846, Santa Cruz Biotechnology), caspase-1 p20 (1:50, cat# SC-398715, Santa Cruz Biotechnology) and NeuN (1:200, cat# MAB377, EMD Millipore, USA) followed by incubation with proper second antibodies. TUNEL staining was performed according to the manufacturer’s instructions (Roche Inc., Indianapolis, USA). Primary neurons were incubated with primary antibody against NeuN (1:200, cat# MAB377, EMD Millipore, USA) and caspase-1 p20 (1:50, cat# SC-398715, Santa Cruz Biotechnology) overnight at 4°C followed by incubation with proper second antibodies. The sections were visualized under a ZEISS HB050 inverted microscope system.

### Nissl Staining

Nissl staining was conducted as previously described (34). In brief, coronal sections were immersed in cresol violet at 37°C for 20 minutes, and then then mounted onto microscope slides with Permount. Staining was visualized under a light microscope and normal neurons with round and palely stained nuclei were counted.

### Neurological Behavior

Neurologic functions were assessed using an 18-point scoring system reported by Sugawara et al (36). Rotarod test was used to assess motor deficits (30). The rotating speed was gradually increased from 4 to 40 rpm over a 5-min period. The latency to fall was recorded. The mean latency was calculated based on three consecutive trials.

### Statistical Analysis

All data were performed with GraphPad Prism 8.02 (GraphPad Software, La Jolla, CA, USA), and expressed as mean ± SD. Differences among multiple groups were compared by one-way analysis of variance with Bonferroni *post hoc* test. Statistical significance was inferred at *P* < 0.05. Linear regression was performed for correlational analysis. Values of *P* < 0.05 was considered statistically significant.

## Results

### EX527 Inhibits SIRT1 Expression, Aggravates Neurological Deficits, and Induces M1 Microglia Polarization and NLRP3 Inflammasome Activation After SAH

**Figure 1:**
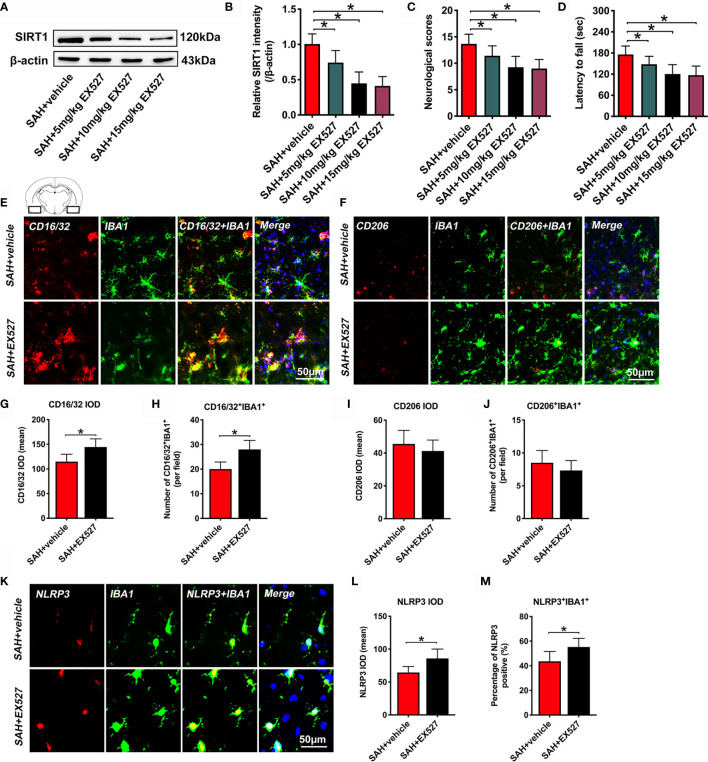
Effects of EX527 on SIRT1 expression, behavior function, microglia polarization, and NLRP3 inflammasome after SAH. Representative western blots **(A)** and quantification of expression of SIRT1 in all groups **(B)**. Quantification of neurological scores **(C)** and rotarod function **(D)** in all groups. Representative immunofluorescence staining images of M1 phenotype **(E)** and M2 phenotype **(F)**, and its quantification analysis **(G–J)**. Representative photomicrographs **(K)** and quantification of NLRP3 immunofluorescence staining **(L, M)**. For histopathology studies, the basal temporal lobe adjacent to the clotted blood was evaluated. Bars represent the mean ± SD. ^*^ *P* < 0.05.

It has been demonstrated that EX527 is a potent and selective SIRT1 inhibitor. We used EX527 pretreatment to mimic SIRT1 knockout in this experiment. We first evaluated the effects of different doses of EX527 on SIRT1 expression. As shown, EX527 dose-dependently decreased SIRT1 expression after SAH (*P* < 0.05) **(** **Figures 1A, B** **)**. In addition, EX527 pretreatment at 5, 10, and 15 mg/kg significantly aggravated neurological outcomes and motor functions after SAH (*P* < 0.05) **(** **Figures 1C, D** **)**. However, there were no significant differences in SIRT1 expression and behavior functions between 10 mg/kg and 15 mg/kg EX527 treatment (*P* > 0.05) **(** **Figures 1A–D**). Thus, we chose 10 mg/kg EX527 for the remaining experiments. We then evaluated the effects of SIRT1 inhibition by EX527 on microglia polarization and NLRP3 inflammasome activation. Our data indicated that EX527 further induced M1 microglia (IBA1^+^/CD16/32^+^) polarization (*P* = 0.0084 for CD16/32 IOD, *P* = 0.0019 for CD16/32^+^IBA1^+^) and NLRP3 inflammasome activation (*P* = 0.0113 for NLRP3 IOD, *P* = 0.023 for NLRP3^+^IBA1^+^), with no statistical difference on M2 microglia (IBA1^+^/CD206^+^) transformation (*P* > 0.05) **(** **Figures 1E–M**).

### SRT1720 Dose-Dependently Enhances SIRT1 Expression, Improves Neurological Functions and Reduces Brain Edema After SAH

**Figure 2:**
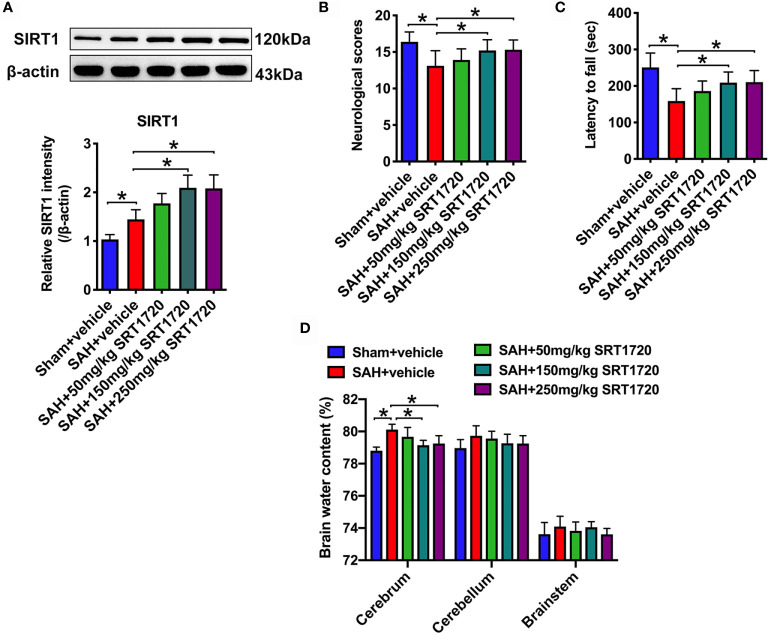
Dose-response effects of SRT1720 on SAH. Representative western blots and quantification of expression of SIRT1 in all groups **(A)**. Quantification of neurological scores **(B)**, rotarod function **(C)**, and brain water content **(D)** in all groups. Bars represent the mean ± SD. ^*^ *P* < 0.05.

SRT1720, a potent SIRT1 activator, was employed in our study. In a dose-response study, SRT1720 was administered to rats after SAH at 50, 150, and 250 mg/kg. Doses of 150 mg/kg and 250 mg/kg (*P* < 0.001, and *P* < 0.001, respectively), but not 50 mg/kg (*P* = 0.1126), markedly increased SIRT1 expression as compared with SAH + vehicle group **(** **Figure 2A** **)**. Additionally, SRT1720 treatment at 150 and 250 mg/kg significantly improved neurologic scores (*P* = 0.0358, and *P* = 0.025, respectively) and rotarod performance (*P* > 0.05, and *P* = 0.0073, respectively), and ameliorated brain water content (*P* = 0.0103, and *P* = 0.0289, respectively) after SAH **(** **Figures 2B–D** **)**. There were no significant differences between 150 and 250 mg/kg SRT1720 in SIRT1 expression (*P* > 0.05), neurological functions (*P* > 0.05) and brain edema (*P* > 0.05) (**Figures 2A–D**). Because 150 mg/kg was the optimum dose to provide maximal effect, we used this dose for the remaining experiments.

### SRT1720 Treatment Activates SIRT1 Signaling and Inhibits Oxidative Stress After SAH

**Figure 3:**
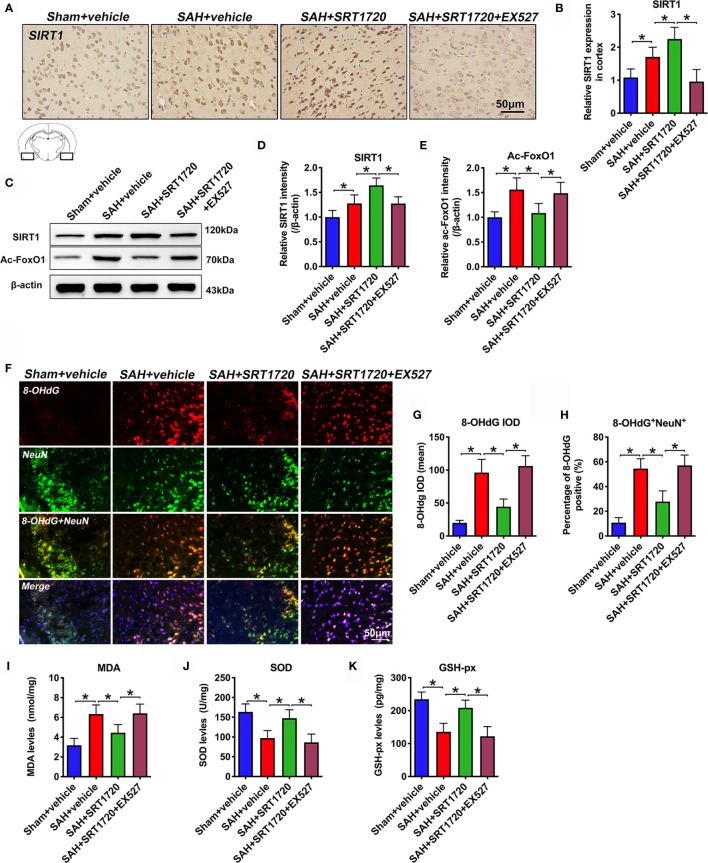
Effects of SRT1720 on SIRT1 activation and oxidative damage after SAH. Representative photomicrographs **(A)** and quantification of SIRT1 immunohistochemistry staining **(B)**. Representative western blots **(C)** and quantification of expressions of SIRT1 **(D)** and ac-FoxO1 **(E)** in all groups. Representative photomicrographs **(F)** and quantification of 8-OHdG immunofluorescence staining **(G, H)**. For histopathology studies, the basal temporal lobe adjacent to the clotted blood was evaluated. Quantification of MDA **(I)**, SOD **(J)**, and GSH-px **(K)** expressions in all groups. Bars represent the mean ± SD. ^*^ *P* < 0.05.

SIRT1 is implicated in a variety of pathophysiological processes, including immune response and redox stress. We first evaluated the effects of SRT1720 on SIRT1 signaling and the subsequent oxidative damage after SAH. Immunohistochemistry showed that the immunoreactivity of SIRT1 was significantly increased after SAH (*P* = 0.0148), which was further enhanced after SRT1720 supplementation (*P* = 0.0388) **(** **Figures 3A, B** **)**. In contrast, EX527 abrogated the enhanced immunoreactivity of SIRT1 by SRT1720 (*P* < 0.001) **(** **Figures 3A, B** **)**. Consistent with the immunohistochemistry data, western blot analysis showed that SRT1720 treatment significantly increased expression of SIRT1 (*P* = 0.0021) and decreased expression of acetyl-FoxO1 (*P* = 0.0026). These changes were all abated by EX527 pretreatment (*P* < 0.05) **(** **Figures 3C–E** **)**. We then evaluated the effects of SRT1720 on oxidative damage after SAH. Double immunofluorescent staining indicated that SRT1720 treatment significantly reduced the 8-OHdG immunity when compared with SAH + vehicle group (*P* < 0.001) **(** **Figures 3F–H**). In addition, SRT1720 administration significantly decreased oxidative insults (*P* = 0.0055) and restored the impairment antioxidant systems after SAH, including SOD and GSH-px levels (*P* = 0.0019, and *P* < 0.001) **(** **Figures 3I–K**). However, EX527 pretreatment reversed the effects of SRT1720 on oxidative damage after SAH (*P* < 0.05) **(** **Figures 3F–H**).

### SRT1720 Treatment Inhibits Inflammatory Response Following SAH

**Figure 4:**
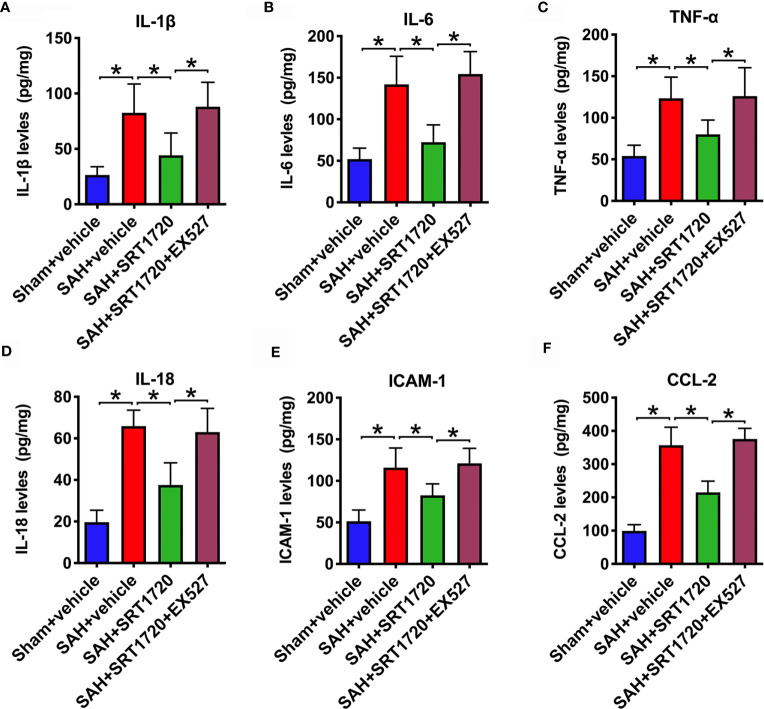
Effects of SRT1720 on inflammatory response after SAH. Quantification of IL-1β **(A)**, IL-6 **(B)**, TNF-α **(C)**, IL-18 **(D)**, ICAM-1 **(E)**, and CCL-2 **(F)** expressions in all groups. Bars represent the mean ± SD. ^*^ *P* < 0.05.

It has been demonstrated that SIRT1 is implicated in the modulation of immune response and could reduce neuroinflammation in a variety of neurological disorders. We then evaluated the effects of SRT1720 on inflammatory response after SAH. As shown, SAH significantly increased IL-1β, IL-6, TNF-α, IL-18, ICAM-1, and CCL-2 (*P* < 0.001) protein levels in the cortex when compared with the sham + vehicle group. SRT1720 treatment markedly reduced these proinflammatory cytokines after SAH (*P* = 0.0172, *P* < 0.001, *P* = 0.0239, *P*< 0.001, *P* = 0.0166, and *P* < 0.001, respectively) **(** **Figures 4A–F**). However, all these changes were abrogated by EX527 pretreatment (*P* < 0.05) **(** **Figures 4A–F**).

### SRT1720 Treatment Modulates M1/M2 Microglia Polarization After SAH

**Figure 5:**
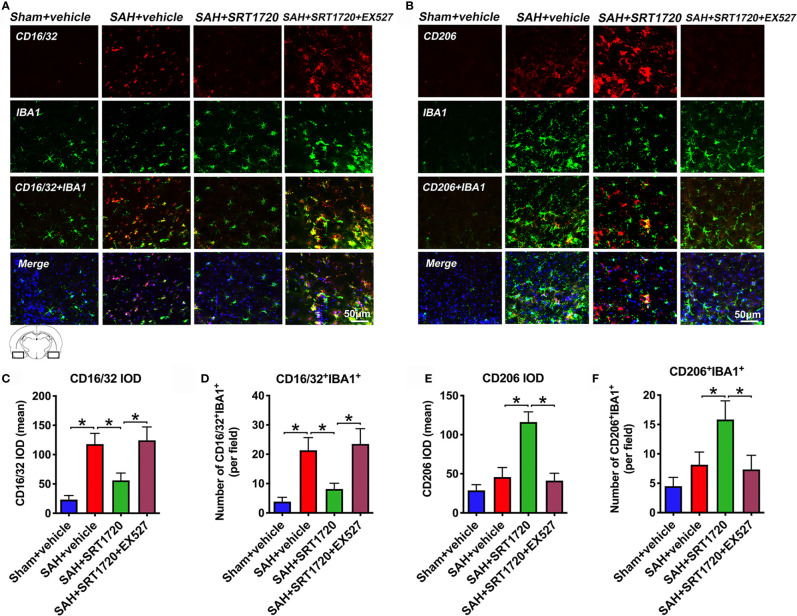
Effects of SRT1720 on microglia phenotypic transformation after SAH. Representative immunofluorescence staining images of M1 phenotype **(A)** and M2 phenotype **(B)**, and its quantification analysis **(C–F)**. For histopathology studies, the basal temporal lobe adjacent to the clotted blood was evaluated. Bars represent the mean ± SD. ^*^ *P* < 0.05.

It has been shown that SIRT1 can modulate the microglia polarization shift from the M1 phenotype and skew toward the M2 phenotype in a variety of disorders. We next evaluated the effects of SIRT1 activation by SRT1720 on M1/M2 microglia polarization following SAH. As shown, the proportion of M1 microglia (IBA1^+^/CD16/32^+^) was significantly increased after SAH as compared with sham group (*P* < 0.001), while treatment with SRT1720 decreased the amount of M1 microglia (*P* < 0.001). Additionally, SRT1720 markedly increased the quantity of M2 microglia (IBA1^+^/CD206^+^) after SAH when compared with SAH + vehicle group (*P* < 0.001) **(** **Figures 5A–F**). However, EX527 pretreatment reversed the effects of SRT1720 on M1/M2 microglia polarization after SAH (*P* < 0.001) **(** **Figures 5A–F**).

### SRT1720 Treatment Inhibits NLRP3 Inflammasome Activation After SAH

**Figure 6:**
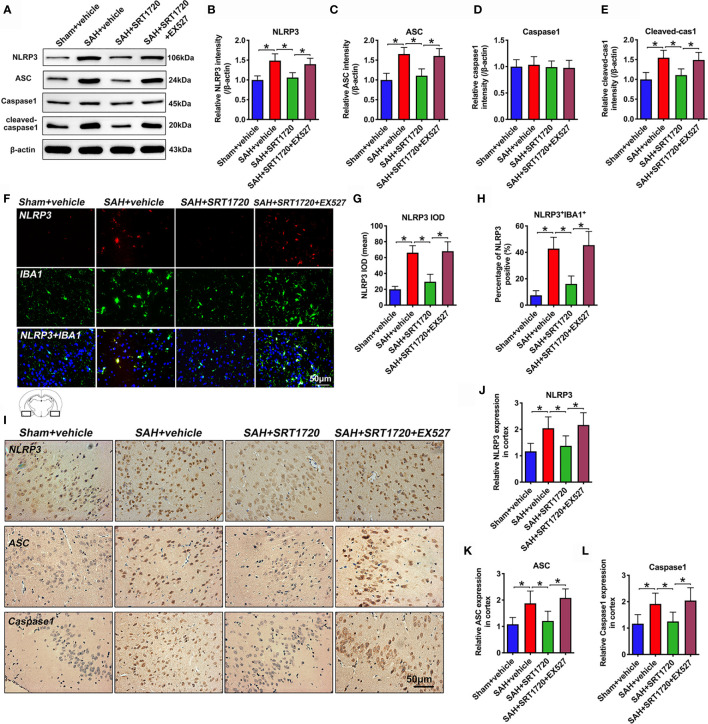
Effects of SRT1720 treatment on NLRP3 inflammasome activation after SAH. Representative western blots **(A)** and quantification of expressions of NLRP3 **(B)**, ASC **(C)**, caspase1 **(D)**, and cleaved caspase1 **(E)** in all groups. Representative photomicrographs **(F)** and quantification of NLRP3 immunofluorescence staining **(G, H)**. Representative photomicrographs **(I)** and quantification of NLRP3 **(J)**, ASC **(K)**, and cleaved caspase-1 **(L)** immunohistochemistry staining. For histopathology studies, the basal temporal lobe adjacent to the clotted blood was evaluated. Bars represent the mean ± SD. Bars represent the mean ± SD. ^*^ *P* < 0.05.

To understand the cellular mechanisms of microglia polarization by SIRT1, we measured NLRP3 inflammasome signaling after SAH. As shown in **Figure 6**, western blot data showed that SAH insults significantly increased the levels of NLRP3, ASC, and cleaved caspase1 (*P* < 0.001) when compared with sham + vehicle group **(** **Figures 6A–E**). SRT1720 treatment markedly decreased the levels of NLRP3 (*P* < 0.001), ASC (*P* < 0.001), and cleaved caspase1 (*P* = 0.002) after SAH, all of which were evidently abated by EX527 pretreatment (*P* < 0.05) **(** **Figures 6A–E**). Double immunofluorescent staining confirmed that SRT720 treatment significantly reduced the expression of NLRP3 in microglia when compared with SAH + vehicle group (*P* < 0.001) **(** **Figures 6F–H**). Meanwhile, immunohistochemistry showed that the immunoreactivities of NLRP3 (*P* = 0.0057), ASC (*P* = 0.0065), and cleaved-caspase-1 (*P* = 0.0198) were significantly increased after SAH, which could be inhibited after SRT1720 supplementation (*P* = 0.0412, *P* = 0.0239, *P* = 0.0425, respectively) **(** **Figures 6I–L**). However, all these changes were prevented by EX527 pretreatment (*P* < 0.05) **(** **Figures 6F–L**). We also performed correlational studies to verify the relationship between SIRT1, NLRP3 inflammasome, and microglia polarization. Our data showed that SIRT1 activation correlated negatively with ROS overproduction (*P* = 0.0127), NLRP3 inflammasome (*P* = 0.0355), and the number of M1 microglia (*P* = 0.0224) (**Supplementary Figure 2A–C**). In contrast, the number of M2 microglia correlated positively with SIRT1 expression (*P* = 0.0341) (**Supplementary Figure 2D**). These data supported that SIRT1 was also involved in the modulation of microglia polarization after SAH.

### SRT1720 Reduces Neuronal Cell Death and Neurological Deficits After SAH

**Figure 7:**
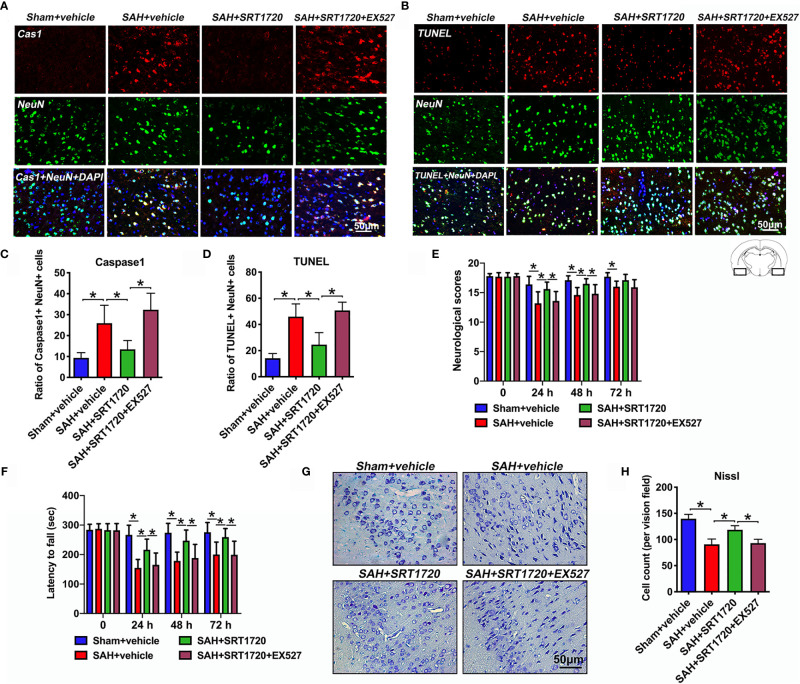
Effects of SRT1720 on neuronal cell death and neurological function after SAH. Representative photomicrographs **(A, B)** and quantification of caspase-1 **(C)** and TUNEL **(D)** staining. For histopathology studies, the basal temporal lobe adjacent to the clotted blood was evaluated. Effects of SRT1720 on neurological scores **(E)** and rotarod performance **(F)**. Representative photomicrographs of Nissl staining **(G)** and quantification of the proportion of surviving neurons **(H)** at 72 h after SAH. Bars represent the mean ± SD. ^*^ *P* < 0.05.

Accumulating evidence indicated that both apoptosis and pyroptosis contribute to the EBI after SAH. Unlike apoptosis, pyroptosis is biochemically characterized as caspase-1 dependent and caspase-3 independent. Notably, suppression of NLPR3 inflammasome or caspase-1 reduces neuronal apoptosis and pyroptosis, and improves neurological outcomes in SAH models. We further explored the effects of SRT1720 on neuronal death and functional outcomes after SAH. As shown, the immunofluorescence staining results showed that there was a low level of caspase-1- and TUNEL-positive neurons in the sham group, which were dramatically increased after SAH (*P* = 0.001, and *P* < 0.001, respectively) **(** **Figures 7A–D**). In contrast, SRT1720 administration could significantly reduce the number of caspase-1- and TUNEL-positive neurons after SAH (*P* = 0.0126, and *P* < 0.001, respectively) **(** **Figures 7A–D**). Concomitant with the reduced neurodegeneration, SRT1720 treatment evidently improved neurological outcomes (*P* < 0.05) and rotarod performance (*P* < 0.05) after SAH **(** **Figures 7E, F** **)**. In addition, Nissl staining showed that SRT1720 treatment significantly improved neuronal survival at 72h after SAH (*P* < 0.001) **(** **Figures 7G, H** **)**. However, all these changes were abated by EX527 pretreatment (*P* < 0.05) **(** **Figures 7A–H**). These indirectly suggested that SRT1720 inhibited NLRP3 inflammasome-mediated neuronal death probably through SIRT1-dependent pathway.

### SRT1720 Ameliorates Inflammatory Response, Oxidative Stress, and Neuronal Damage *In Vitro*

**Figure 8:**
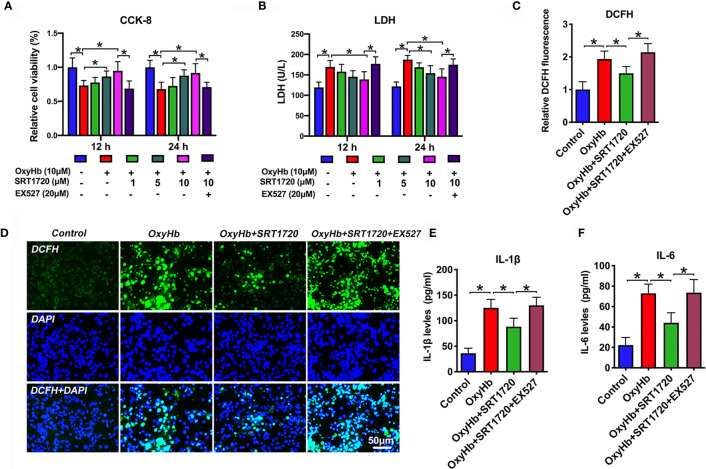
Effects of SRT1720 treatment on oxidative stress, inflammatory response, and neuronal damage *in vitro*. Quantitative analysis of CCK-8 **(A)** and LDH activity **(B)** in the indicated groups. Quantification **(C)** and representative photomicrographs **(D)** of DCFH immunofluorescence in primary cortical neurons. Quantitative analysis of IL-1β **(E)**, and IL-6 **(F)** in the indicated groups. Bars represent the mean ± SD. ^*^ *P* < 0.05.

To confirm the proposed crosstalk between microglial and neuronal cells, a transwell co-culture system was established *in vitro*. We first evaluated whether SRT1720 had beneficial effects *in vitro* SAH model. As shown, primary neurons and microglia stimulated with OxyHb induced a marked decrease in neuronal viability (*P* < 0.05), and increase in ROS generation (*P* < 0.001) and proinflammatory cytokines including IL-1β (*P* < 0.001) and IL-6 (*P* < 0.001), all of which were reversed by SRT1720 treatment (*P* < 0.05) **(** **Figures 8A–F**). In contrast, EX527 abrogated the beneficial effects of SRT1720 on oxidative stress, inflammatory response, and neuronal damage (*P* < 0.05) **(** **Figures 8A–F**). Consistent with the *in vivo* results, our findings indicated that SRT1720 could exert a cerebroprotective action against SAH damage *in vitro*.

### SRT1720 Enhances SIRT1 Activation and Inhibits NLRP3 Inflammasome *In Vitro*

**Figure 9:**
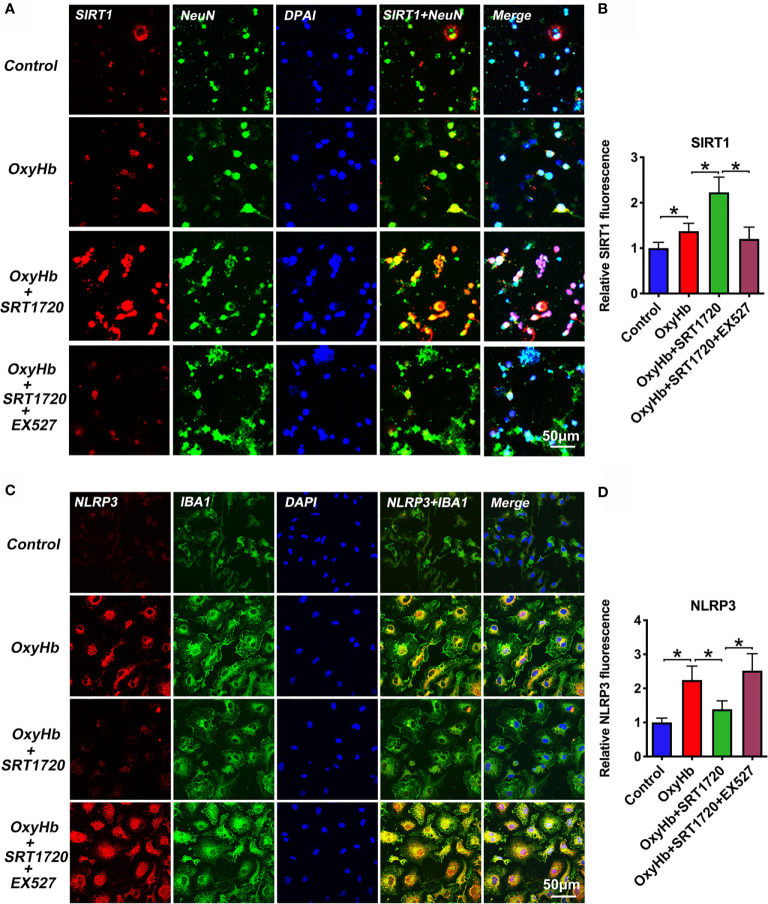
Effects of SRT1720 treatment on SIRT1 and NLRP3 staining *in vitro*. Representative photomicrographs **(A)** and quantification **(B)** of SIRT1 staining in primary cortical neurons. Representative photomicrographs **(C)** and quantification **(D)** of NLRP3 staining in primary microglia. Bars represent the mean ± SD. ^*^ *P* < 0.05.

To further explore the mechanisms of action of SRT1720 *in vitro* SAH model, we examined the possible effects of SRT1720 on SIRT1 and NLRP3 expression in neuron-microglia co-cultures by using double immunofluorescence staining. At the cellular level, SIRT1 is known as a nuclear protein, which is predominantly expressed in neurons. NLRP3 inflammasome is mainly expressed in microglia and the basal level of the NLRP3 inflammasome is low under physiological conditions. Consistent with previous studies, our immunofluorescence staining data indicated that SIRT1 was weakly expressed in primary neurons and the immunoreactivity of NLRP3 was low in primary microglia in the control group. OxyHb stimulation induced the expression of SIRT1 (*P* = 0.0397) and NLRP3 (*P* < 0.001) in the co-culture system. SRT1720 treatment could significantly enhance the expression of SIRT1 (*P* < 0.001) in primary cortical neurons and decrease NLRP3 expression (*P* = 0.002) in primary microglia, which could be abrogated by EX527 administration (*P* < 0.05) **(** **Figures 9A–D**). These data consistent with the results *in vivo* suggesting that SIRT1 could modulate NLRP3 inflammasome activation and SRT1720 treatment was able to activate SIRT1 and inhibit NLRP3 inflammasome signaling.

### SRT1720 Decreases Neuronal Pyroptosis and Apoptosis After SAH *In Vitro*

**Figure 10:**
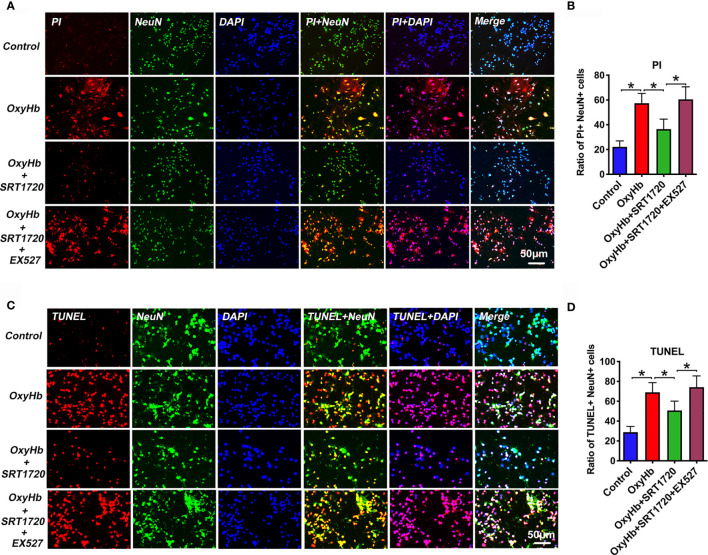
Effects of SRT1720 on PI and TUNEL staining *in vitro*. Representative photomicrographs **(A)** and quantification **(B)** of PI staining in primary cortical neurons. Representative photomicrographs **(C)** and quantification **(D)** of TUNEL staining in primary cortical neurons. Bars represent the mean ± SD. ^*^ *P* < 0.05.

Both neuronal apoptosis and pyroptosis are involved in the pathophysiology of EBI after SAH. Since PI cannot pass intact plasma membrane and can only be present in DNA of cells where the plasma membrane has been injured. During pyroptosis, pores can be formed in the cell membrane and can be detected by PI staining [26]. Therefore, PI staining is widely used as a detection for neuronal pyroptosis. In our study, we performed PI and TUNEL staining to examine neuronal pyroptosis and apoptosis, respectively. Based on the data *in vivo*, we speculated that SIRT1 activation by SRT1720 could reduce neuronal apoptosis and pyroptosis *in vitro*. As shown, double immunofluorescence staining showed that PI- (*P* < 0.001) and TUNEL-positive (*P* < 0.001) neurons were significantly increased after OxyHb incubation, which could be decreased by SRT1720 administration (*P* = 0.0011, and *P* = 0.013, respectively) **(** **Figures 10A–D**). These confirmed that SRT1720 was able to reduce neuronal pyroptosis and apoptosis after SAH *in vitro*. In contrast, EX527 abated the protective effects of SRT1720 on neuronal cell death *in vitro* (*P* < 0.05) **(** **Figures 10A–D**). These findings indirectly indicated that SIRT1 activation by SRT1720 probably inhibited NLRP3 inflammasome-mediated pyroptosis and apoptosis.

## Discussion

In this study, we showed that EX527 significantly inhibited SIRT1 expression, aggravated neurological outcomes and motor functions after SAH. In the meantime, EX527 pretreatment induced M1 microglia polarization and NLRP3 inflammasome activation after SAH. SIRT1 activation by SRT1720 exerted a neuroprotective effect after SAH. The application of SRT1720 significantly ameliorated neurological deficits, brain edema, oxidative damage, neuroinflammation, and neural cell death after SAH. In addition, SIRT1 activation by SRT1720 decreased the number of M1 microglia and upregulated the expression of M2 microglia, leading to improved SAH recovery. Furthermore, EX527 abated the beneficial effects of SRT1720 against SAH both *in vivo* and *in vitro*. Meanwhile, we confirmed that ROS-mediated NLRP3 inflammasome signaling might be involved in the beneficial effects of SRT1720 on microglia polarization after SAH.

Cerebral inflammation is a vital component of the pathological cascade in the EBI after SAH (12). Microglia, as the resident immunocytes of the central nervous system (CNS), react to various brain injury and can be activated by blood components, including red cells, and heme (37). It has been demonstrated that microglia respond to acute brain injury by becoming activated and developing classic M1-like or alternative M2-like phenotypes. M1 microglia are the primary source of cytokines, chemokines, ROS, and other immunomodulatory molecules, which further increase brain damage. In contrast, M2 microglia are considered as nerve repair cells, as they produce anti-inflammatory factors and upregulate neuroprotective factors in neurological disorders (6, 37). Though the polarization of microglia is controversial, the dichotomy between M1 and M2 phenotypes classification remains useful for clarifying the character of microglia in various brain disorders (4, 38). In models of TBI, spinal cord injury, ischemic stroke, and intracerebral hemorrhage, an M2 to M1 shift was observed in the early period (6, 8, 37). In time, the M1 microglia/macrophages become dominant in the injury area and aggravate brain damage by exacerbating inflammatory response. Similarly, in SAH area, the phenomenon of microglia polarization has also been reported. Mounting evidence has indicated that inhibition of microglia M1 polarization and promotion of a shift from M1 to M2 phenotype could ameliorate EBI and improve neurological functions after SAH (10–12).

SIRT1 is a member of NAD^+^-dependent protein deacetylases implicated in a variety of pathophysiological processes, including immune response and redox stress (13, 15). Multiple studies have demonstrated that SIRT1 exerts a protective role in various models of neuronal injury and neurodegeneration. For example, Vellimana et al. reported that SIRT1 activation by resveratrol-conditioning provided cerebroprotection effects after cerebral ischemia at both early and delayed time points (20). Hattori et al. demonstrated that SIRT1 overexpression upregulates the nitric oxide system and counters cerebral hypoperfusion injury (39). In SAH area, accumulating studies also indicate that SIRT1 activation could attenuate EBI and delayed cerebral vasospasm, and inhibition of SIRT1 could aggravate SAH-induced vascular dysfunction and functional deficits (3, 20, 31, 40). These suggest that SIRT1 might represent a potential therapeutic target for treating SAH. However, to data, little is known about the potential effects of SIRT1 on microglia polarization and the underlying molecular mechanisms after SAH.

According to current literature, SIRT1 can modulate the microglia polarization shift from the M1 phenotype and skew toward the M2 phenotype in a variety of disorders (22, 24, 33, 41). A previous study by Chen et al. reported that SIRT1 activation by SRT1720 significantly improved functional recovery after SCI by reducing the levels of proinflammatory cytokines, and the number of M1 macrophages (33). Fu et al. indicated that SIRT1 activation could modulate the microglia polarization shift from the M1 phenotype and skew toward the M2 phenotype in a cerebral ischemic/reperfusion injury model (24). Thus, we speculated that SIRT1 might modulate microglial phenotypic transformation to protect against EBI after SAH. In our study, we first employed EX527, a potent and selective SIRT1 inhibitor, to suppress SIRT1 to mimic SIRT1 knockout. We observed that EX527 significantly inhibited SIRT1 activation and promoted microglia phenotype toward M1 after SAH. To further confirm whether SIRT1 modulates microglia polarization, a specific activator of SIRT1 SRT1720 was used. SRT1720 is reported as a potent SIRT1 activator with 1000-fold efficacy compared to resveratrol. Multiple studies have demonstrated that SRT1720 can effectively activate SIRT1 expression and activation in a variety of diseases models (33, 42). In the CNS diseases, SRT1720 has been proved to ameliorate neuronal injury and improve functional recovery in many neurological disorders (43, 44). Moreover, SRT1720 can cross the blood-brain barrier and act directly on the CNS (33). In our study, we confirmed that SRT1720 significantly increased SIRT1 activation and improved locomotor recovery after SAH. Additionally, we observed that the number of M1 microglia was significantly decreased after SRT1720 treatment, while the percentage of M2 microglia was increased. Our data suggested that SIRT1 activation by SRT1720 is able to regulate microglia M1/M2 polarization after SAH, which may explain the observed locomotor recovery after injury. However, the mechanisms of microglial M1-to-M2 phenotype transition by SRT1 activation remain unknown.

The NLRP3 inflammasome is a cytoplasmic multiprotein complex of the innate immune system that can positively trigger a series of proinflammatory pathways. Mounting evidence has demonstrated that NLRP3 inflammasome is involved in microglia-induced inflammatory responses and microglial polarization (26, 27). Chen et al. reported that MitoQ decreased the expression of M1 markers and increased the expression of M2 markers both *in vivo* and *in vitro* after intracerebral hemorrhage through inhibiting the ROS/NLRP3 inflammasome pathway. Moreover, NLRP3 siRNA shifted FeCl_2_-treated microglia from the M1 to the M2 cells (45). In a stress-induced hypertension rat model, prorenin increased the ROS-triggering M1 phenotype-switching and NLRP3 activation, while NLRP3 inhibitor MCC950 decreased the M1 polarization in the rostral ventrolateral medulla (46). In SAH studies, accumulating evidence has indicated that NLRP3 is the main participant in neuroinflammation and inhibition NLRP3 could ameliorate EBI and delayed cerebral vasospasm after SAH (47, 48). Upon stimulation by a wide range of signals including ROS, the NLRP3 inflammasome is assembled and activated to trigger caspase-1 activation. The activated caspase-1 can proteolytically cleave pro-inflammatory cytokines into their active forms amplifying the innate immune response. Additionally, activated caspase-1 could induce pyroptosis to further aggravate neural cell death. According to recent studies, SIRT1 is able to inhibit NLRP3 inflammasome activation in different research fields. Shaheen et al. reported that saffron attenuated neuroinflammation in TBI mouse model by suppressing NLRP3 inflammasome *via* SIRT1 activation (49). Li et al. indicated that dioscin provided protection against SAH *via* the suppression of NLRP3 inflammasome through SIRT1-dependent pathway (3). However, how SIRT1 regulates NLRP3 inflammasome inhibition remains unclear. A possible mechanism might involve ROS production (29, 50).

ROS can elicit a plethora of detrimental effects on cellular functions by causing damages to proteins, lipids and nucleic acids (51). Neurons can generate elevated amounts of ROS compared to other organs and are particularly vulnerable to ROS (52). It is known that both ROS overproduction and neuroinflammation are considered crucial elements of EBI after SAH and each of them promotes and amplifies the other one (3, 53). Additionally, ROS production is one of the major signals that trigger the NLRP3 inflammasome activation (54). According to previous studies, SIRT1 is predominantly expressed in neurons and the elevated SIRT1 activation could significantly reduce oxidative damage by deacetylating FoxOs (13, 31). As transcription factors, FoxOs play a critical role in the maintenance of intracellular ROS homeostasis. FoxO1, a member of the FoxOs, could transcriptionally induce numerous antioxidative genes to protect against oxidative damage. It has been demonstrated that overexpression of FoxO1 can strengthen H_2_O_2_ scavenging and oxidative stress resistance, while decreased FoxO1 could aggravate ROS overproduction and oxidative insults (55). As an upstream mediator of FoxO1, SIRT1 could deacetylate FoxO1 to enhance FoxO1 DNA binding and induce the expression of FoxO1 target genes to mitigate oxidative stress in a variety of disorders (31, 55). Consistent with previous studies, we observed that SIRT1 activation by SRT1720 significantly increased deacetylation of FoxO1, ameliorated the overproduction of ROS and inhibited NLRP3 inflammasome activation after SAH. Concomitant with the suppression of ROS-NLRP3 inflammasome pathway, SRT1720 ameliorated SAH-induced functional deficits and neuronal apoptosis and pyroptosis. In contrast, EX527 pretreatment abated the inhibitory effects of SRT1720 on ROS-NLRP3 inflammasome signaling and the neuroprotective effect in SAH. Similarly, *in vitro*, SIRT1 activation by SRT1720 ameliorated neuroinflammation, oxidative damage, and neuronal damage in neurons and microglia co-culture system stimulated with OxyHb, which were associated with ROS-NLRP3 inflammasome suppression, and were counteracted by EX527. Taken together, SIRT1 inhibits neuroinflammation by reduction in M1 microglia polarization and driving the microglial phenotype toward M2, which might be mediated by modulation of the ROS-NLRP3 inflammasome signaling pathway.

Driving microglia M2 polarization and maintaining the balance of M1/M2 should be of great significance for the treatment of acute brain injuries. However, most of compounds discovered can only suppress inflammation by decreasing M1 phenotype microglia, and only a few compounds can promote microglia M2 polarization. This study showed that SIRT1 activation can significantly decrease the M1 phenotype microglia and promote the M2 polarization of microglia, which might explain the observed neuroprotection. However, we have to admit that there are several limitations in our study. Firstly, EX527 may not act as a highly specific inhibitor of SIRT1. EX527 can also slightly affect SIRT2 and SIRT3 pathway. Additionally, SRT1720 can affect other SIRTs members including SIRT3. Thus, SIRT1 gene knockout mice and SIRT1 overexpression by transfection of adeno-associated virus should be performed in the future to confirm our investigations. Secondly, the long-term inflammation also plays an important role in the pathophysiology after SAH (56). We did not evaluate the potential effects of SIRT1 on the long-term neuroinflammation. In our future studies, the endovascular puncture SAH model with heavier brain damage will be further employed. Lastly, it has reported that M2 microglia could divide into multiple phenotypes, including M2a, M2b, and M2c (6). In our study, we did not explore the functionality of the various M2 subtypes after SRT1720 treatment. Therefore, more pre-clinical studies are required to decipher these issues.

## Conclusion

In summary, we provide the first preclinical evidence that SIRT1 activation by SRT1720 might have dramatic effects on inflammation and microglial polarization after SAH. These effects were at least in part through inhibiting ROS-mediated NLRP3 inflammasome signaling. Thus, SIRT1 might be a promising drug target for SAH treatment and the SIRT1 agonist SRT1720 may have great potential for SAH.

## Data Availability Statement

The data that support the findings of this study are available from the corresponding author upon reasonable request. Some data may not be made available because of privacy or ethical restrictions.

## Ethics Statement

All procedures were approved by the Animal Care and Use Committee of Wannan Medical College.

## Author Contributions

D-YX, J-LY, X-CJ, and MQ performed the *in vivo* studies. D-YX, N-SL, and L-YW performed the *in vitro* studies. D-YX, L-YW, and X-SZ contributed to the design and analysis of the study and revised the manuscript. All authors analyzed the results and approved the final version of the manuscript. All authors have read and agreed to the published version of the manuscript.

## Funding

This work was supported by grants from The National Natural Science Foundation of China (No. 81801166), Fundamental Research Funds for the Central Universities (No. 14380478), Jiangsu Provincial Double-Innovation Doctor Program for L-YW, The Science Research Project of Professional of the First Affiliated Hospital of Wannan Medical College (No. YR201911), and Domestic Visiting Scholar Program for Excellent Young Talents in Colleges and Universities of Anhui Province (No. gxgnfx2021125).

## Conflict of Interest

The authors declare that the research was conducted in the absence of any commercial or financial relationships that could be construed as a potential conflict of interest.

## Publisher’s Note

All claims expressed in this article are solely those of the authors and do not necessarily represent those of their affiliated organizations, or those of the publisher, the editors and the reviewers. Any product that may be evaluated in this article, or claim that may be made by its manufacturer, is not guaranteed or endorsed by the publisher.

## Supplementary Material

Supplementary Figure 1 Immunofluorescence staining showed the purity of primary microglia was more than 90%.

Supplementary Figure 2 Correlation of ROS production **(A)**, NLRP3 inflammasome **(B)**, and the number of M1 microglia **(C)** and M2 microglia **(D)** with SIRT1 expression in the SAH + SRT1720 and SAH+SRT1720+EX527 groups. Scatter plot shows SIRT1 expression correlated negatively with ROS production (*P* < 0.05), NLRP3 inflammasome (*P* < 0.05), and the number of M1 microglia (*P* < 0.05). In contrast, the number of M2 microglia correlated positively with SIRT1 expression (*P* < 0.05).
