# Supplementary figures and images for: SIRT1 Promotes M2 Microglia Polarization via Reducing ROS-Mediated NLRP3 Inflammasome Signaling After Subarachnoid Hemorrhage
Source: Front Immunol. 2021 Nov 24;12:770744. doi: 10.3389/fimmu.2021.770744 (PMC8653696; doi:10.3389/fimmu.2021.770744)

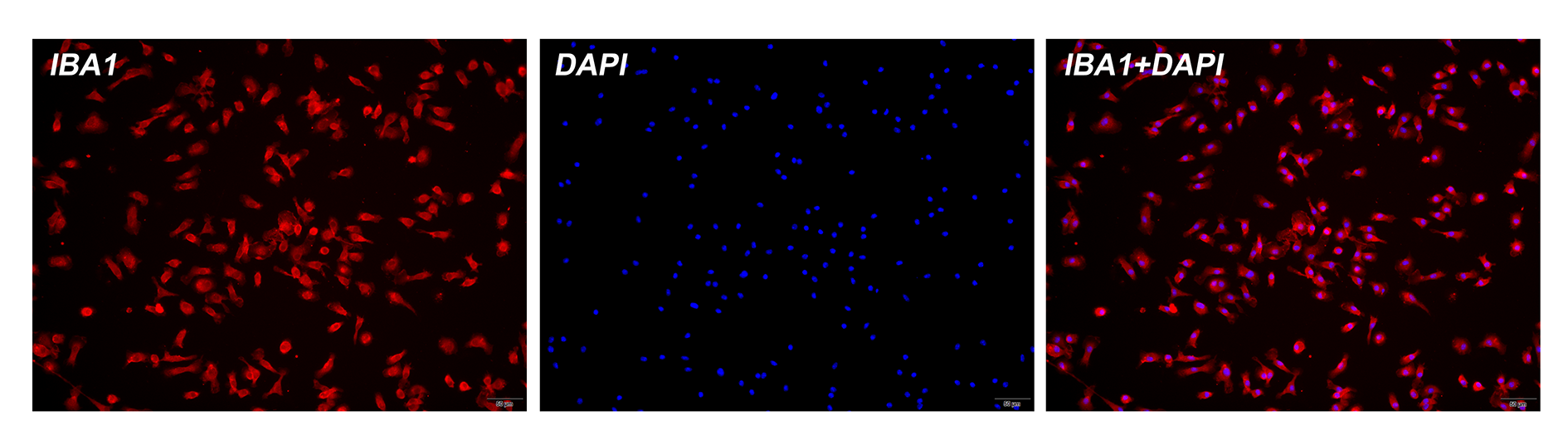

Supplement: Supplementary Figure 1 — Immunofluorescence staining showed the purity of primary microglia was more than 90%. [file Image_1.tif]

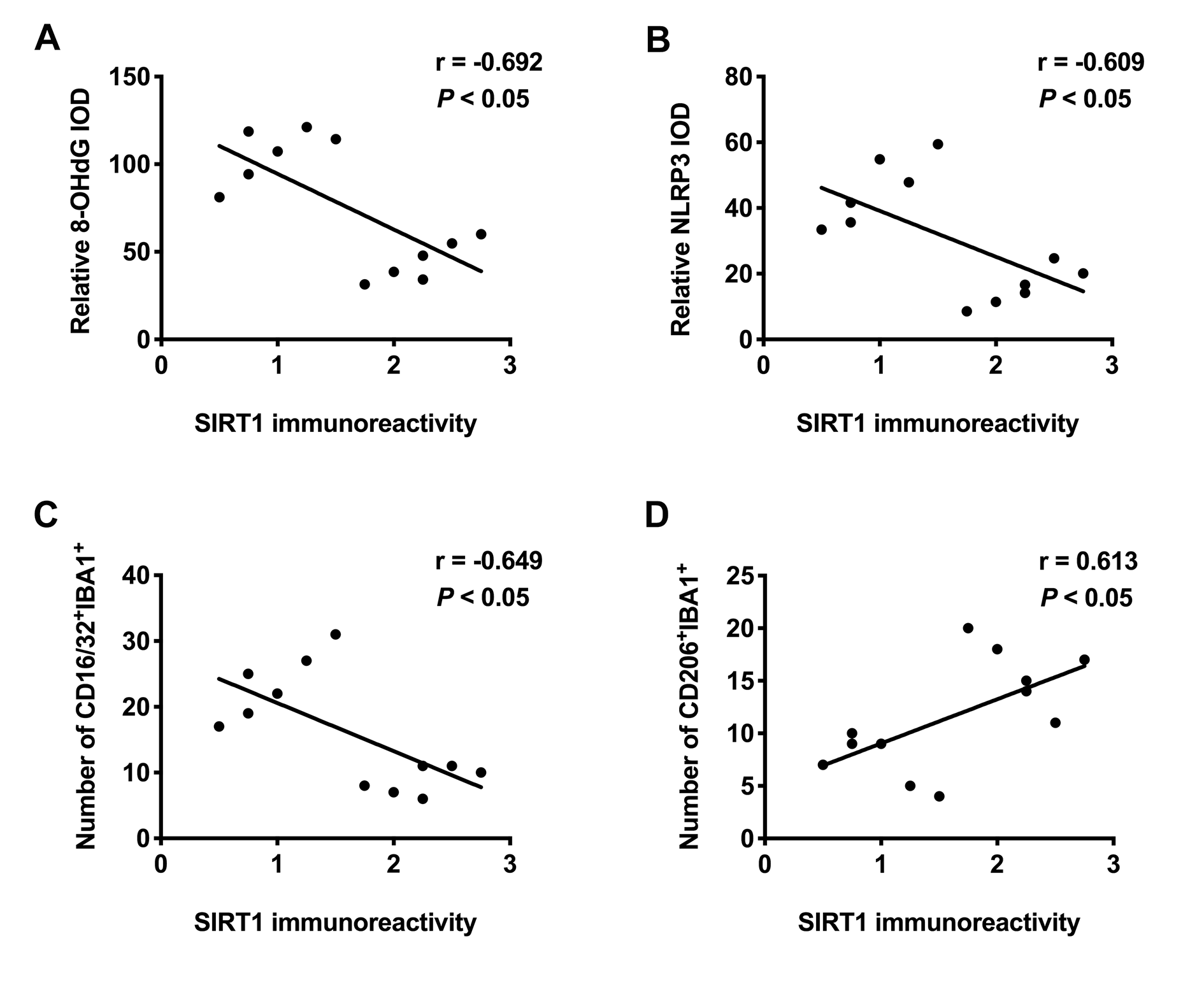

Supplement: Supplementary Figure 2 — Correlation of ROS production (A), NLRP3 inflammasome (B), and the number of M1 microglia (C) and M2 microglia (D) with SIRT1 expression in the SAH + SRT1720 and SAH+SRT1720+EX527 groups. Scatter plot shows SIRT1 expression correlated negatively with ROS production (P < 0.05), NLRP3 inflammasome (P < 0.05), and the number of M1 microglia (P < 0.05). In contrast, the number of M2 microglia correlated positively with SIRT1 expression (P < 0.05). [file Image_2.tif]
